# Long-Term Immunogenicity upon Pertussis Booster Vaccination in Young Adults and Children in Relation to Priming Vaccinations in Infancy

**DOI:** 10.3390/vaccines10050693

**Published:** 2022-04-28

**Authors:** Pauline Versteegen, Axel A. Bonačić Marinović, Pieter G. M. van Gageldonk, Saskia van der Lee, Lotte H. Hendrikx, Elisabeth A. M. Sanders, Guy A. M. Berbers, Anne-Marie Buisman

**Affiliations:** 1 National Institute for Public Health and the Environment, Centre for Infectious Disease Control, P.O. Box 1, 3720 BA Bilthoven, The Netherlands; axel.bonacic.marinovic@rivm.nl (A.A.B.M.); pieter.van.gageldonk@rivm.nl (P.G.M.v.G.); VIKING@rivm.nl (S.v.d.L.); lieke.sanders@rivm.nl (E.A.M.S.); guy.berbers@rivm.nl (G.A.M.B.); 2 Public Health Service (GGD) of Utrecht, Department of Corona Vaccinations, P.O. Box 51, 3700 AB Zeist, The Netherlands; 3 Department of Paediatric Immunology and Infectious Diseases, Wilhelmina Children’s Hospital, Lundlaan 6, 3584 EA Utrecht, The Netherlands; 4 Department of Paediatrics, Bovenij Hospital, P.O. Box 37610, 1030 BD Amsterdam, The Netherlands; l.hendrikx@bovenij.nl

**Keywords:** pertussis, vaccination, serology, infection, human studies

## Abstract

Booster vaccinations for pertussis are advised in many countries during childhood or adulthood. In a phase IV longitudinal interventional study, we assessed long-term immunity following an extra pertussis booster vaccination in children and adults. Children (9 years of age) were primed in infancy with either the Dutch whole cell pertussis (wP) vaccine (*n* = 49) or acellular pertussis (aP) vaccines (*n* = 59), and all children received a preschool aP booster. Adults (25–29 years, *n* = 86) were wP-primed in infancy and did not receive a preschool booster. All were followed-up for approximately 6 years. After the additional booster, antibody responses to pertussis were more heterogeneous but generally higher in adults compared with children, and additional modelling showed that antibody concentrations remained higher for at least a decade. Serologic parameters indicative of recent pertussis infection were more often found in aP-primed children (12%) compared with wP-primed individuals (2%) (*p* = 0.052). This suggests that the aP booster vaccination in aP-primed children offers less long-term protection against pertussis infection and consequently against transmission. Together, these data show that aP priming in combination with aP boosting may not be sufficient to prevent circulation and transmission, while wP-primed adults may benefit from enhanced long-lasting immunity.

## 1. Introduction

Pertussis is an infectious disease that may have a severe course and can be life-threatening, particularly in infancy [1]. Older adults and individuals with pulmonary comorbidities are prone to its complications [2]. The introduction of a whole cell pertussis (wP) vaccine in the 1940–1950s almost completely eliminated pertussis cases in countries with high vaccination coverage during the first decades after implementation [3]. Although global pertussis incidence is still decreasing, a resurgence in pertussis has been seen in many countries over recent decades, despite high vaccine coverage [4]. In the Netherlands, an increase in pertussis reporting began in 1996, presumably following the introduction, in the early 1990s, of a Dutch wP vaccine with low effectiveness [5,6]. Since then, several changes in the Dutch national immunisation programme (NIP) have been made (Table S1). Currently, acellular pertussis (aP) immunisations are advised at 3, 5, and 11 months of age and at 4 years of age but not for school aged children or adults.

Because of the reactogenicity profile of wP vaccines, many high-income countries switched to aP vaccines when these became available in the 1990s [7,8]. Next to the lower reactogenicity, the short-term immunogenicity based on humoral responses of the aP vaccines seemed superior compared with wP vaccines. Following the switch to aP vaccines, however, an increase in pertussis incidence was noted in many countries [9]. The Netherlands switched from wP to aP vaccines in 2005 and later observed an increase in reported disease incidence [6,8]. The increase was observed in children, adolescents, and adults who can serve as an important reservoir of pertussis circulation and may be the source of transmission to infants who need to be protected to avoid severe disease or death [10]. For this reason, many countries added aP booster doses not only at preschool age, but also for school-aged children, adolescents, adults, and older adults or specific target groups such as pregnant women, healthcare workers, and military conscripts [11,12,13,14].

Long-term immunogenicity studies that compare adolescent and adult immune responses to aP booster vaccinations are rare and in general show similar antibody kinetics [15,16]. However, some differences have been reported, with initially higher humeral responses against pertussis toxin (Ptx) in adolescents, while follow-up antibody concentrations against pertactin (Prn) were higher in adults. In both adolescents and adults, Ptx-specific antibody concentrations declined to almost pre-vaccination levels within 5 years, although in a prediction model, adult long-term antibody concentrations would remain above a presumed protective level of 20 international units (IU)/mL for over 9 years after aP booster vaccination in the case of wP-primed young adults [17]. In paediatric immunogenicity studies, aP- and wP-priming in infancy was compared after an aP booster vaccination at 4 years and an additional booster at 9 years of age. Higher Ptx and filamentous haemagglutinin (FHA) immunoglobulin (Ig) G antibody concentrations one month post the additional aP booster vaccination in wP-primed children at age 9 years were reported [18]. The differences in Ptx and FHA IgG had disappeared one year post-booster vaccination.

Here we investigate differences in the long-term pertussis antibody concentrations after an additional aP booster vaccination at 9 years of age comparing aP- and wP-primed children. With respect to age, wP-primed children and wP-primed adults 25–29 years of age were followed-up until 6 years post the additional aP booster vaccination. These types of data provide information for vaccine policy concerning additional booster vaccinations in the current Dutch NIP to reduce infection, transmission, and the disease burden of *Bordetella pertussis* in the Netherlands.

Our data implies that extra aP pertussis booster vaccinations for aP-primed children offer only limited protection against infection and circulation of *B. pertussis*.

## 2. Materials and Methods

### 2.1. Study Design and Participants

**Figure 1:**
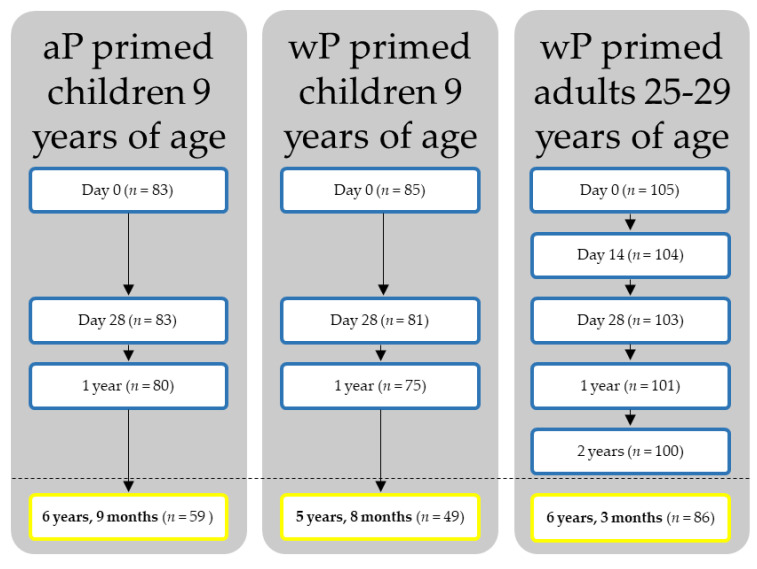
Flow scheme. aP: acellular pertussis; wP: whole cell pertussis.

Two groups of healthy children and one group of adult participants included in this immunology study originated from three different interventional cohort studies in the Netherlands as previously described, with the flow scheme presented in Figure 1 [17,18,19]. For the present long-term follow-up study, a single additional sampling timepoint around 6 years post the study aP booster vaccination was added in all three study cohorts. Paediatric study participants were routinely primed in infancy with either the Dutch wP vaccine or different aP vaccines at 2, 3, 4 and 11 months of age. All children were aP-boosted at 4 years, and at 9 years of age received the additional study booster vaccine containing diphtheria toxoid, tetanus toxoid, pertussis toxoid, filamentous haemagglutinin, pertactin, and inactivated poliovirus (dTap3-IPV, Boostrix-IPV, GlaxoSmithKline (GSK), Rixensart, Belgium). This was in 2009 for wP-primed children and in 2013 for aP-primed children. Results of previous studies at baseline, 28 days post-vaccination and one-year post-vaccination are reported [18,19]. For the present study, a single additional blood sample was drawn by venepuncture 5 years and 8 months post-vaccination in wP-primed children (study number 2013-001864-50/NTR4089) and by fingerprick 6 years and 9 months post-vaccination in the aP-primed children (study numberISRCTN644117538) [18,19]. In addition, young adults, who were primed in infancy with the Dutch wP vaccine at 3, 4, 5 and 11 months without a booster at 4 years, received the study dTap3 booster (Boostrix, GSK) in 2014 at age 25–29 years. Data at baseline, day 14, day 28, 1 year, and 2 years post the aP booster vaccination are published [17]. For the current study, a single additional blood sample was collected by fingerprick 6 years and 3 months post the booster vaccination in these adults (study number 2013-005355-32/NTR4494) [17]. An overview of timelines and recruitment regions is provided in Figure S1, and the cumulative local disease notifications in the included regions at times of the studies can be viewed in Figure S2. All participants filled out a questionnaire at the last timepoint concerning information regarding self-reported, laboratory confirmed and/or clinically treated pertussis infection during the study and regarding extra pertussis vaccinations (i.e., a maternal aP booster) during the follow-up period (in addition to the study booster vaccination).

### 2.2. Serological Analysis

The small volumes obtained per participant were sufficient to measure serum IgG concentrations against Ptx (Netherlands Vaccine Institute (NVI)), Bilthoven, Netherlands), FHA (Kaketsuken, Kumamoto, Japan), Prn [20], diphtheria toxoid (Dtxd) (NVI), and tetanus toxin (Ttx) (T3194, Sigma Aldrich, Saint Louis, MO, USA) and were quantified using a fluorescent-bead-based multiplex immunoassay (MIA) with independent duplicates, as previously described [21,22].

Briefly, the conjugated fluorescent microbeads were incubated with plasma or serum samples in two dilutions (200 and 4000), and a reference serum in a dilution series, and control sera on each plate. The measurement was performed using a BioPlex LX 200 combined with BioPlex Manager 6.2 (Bio-Rad Laboratories, Hercules, CA, USA). To express antibody concentrations in international units (IU)/mL, an in-house standard, calibrated on the pertussis antiserum (human) 1st WHO International Standard, was used. For each analyte, the mean fluorescent intensity was converted to IU/mL by interpolation from a five-parameter logistic standard curve. The lower limit of quantification (LLOQs) was 0.85 IU/mL for Ptx, 0.82 IU/mL for FHA, 1.0 IU/mL for Prn, 0.001 IU/mL for Dtxd, and 0.001 IU/mL for Ttx. For Dtxd and Ttx, 0.1 IU/mL was defined as the cut-off of protection [23,24]. For IgG-Ptx, 20 IU/mL was defined as arbitrary cut-off for protection against clinical disease [25,26,27]. Pertussis infection at baseline was defined as IgG-Ptx ≥100 IU/mL in the absence of a recent vaccination which conforms to the Dutch pertussis infection diagnostic criterion using a single serum/plasma sample [28]. Infection during the study was defined as a three-fold increase in IgG-Ptx between the 1-year timepoint and the last timepoint, combined with IgG-Ptx ≥20 IU/mL at the last timepoint, which conforms to the Dutch pertussis infection diagnostic criterion using a paired serum/plasma sample [28].

### 2.3. Stastical Analyses

The primary outcome is the difference in IgG antibody concentration kinetics between the age groups and between the different priming vaccination backgrounds for pertussis toxin (Ptx), filamentous haemagglutinin (FHA), pertactin (Prn), diphtheria toxoid (Dtxd), and tetanus toxin (Ttx).

At all study timepoints, geometric mean concentrations (GMCs) were calculated for the three cohorts. Additionally, the GMCs at the last timepoints were separately calculated for (1) participants who developed a pertussis infection during the study based on antibody concentrations as indicated in the serological analysis, (2) participants who received an extra vaccination during the study (in addition to the study intervention) indicated by the questionnaire, and (3) participants who did not get infected nor received an extra pertussis vaccine during the study. One participant was excluded from all analyses because antibody concentrations on multiple antigens did not match the questionnaire nor the patterns of the different antigens measured.

Predictions from a bi-exponential IgG antibody decay model was used to compare antibody decay rates among the cohorts [29,30,31]. This model gave insight in kinetics and offered us the possibility to calculate the estimated duration of protection. The model was fitted under a Bayesian statistical framework to the participant antibody data by using Markov chain Monte Carlo simulations run with the sampling software JAGS, and interfaced with the R Statistical Software [32,33]. Pertussis antigen data from participants with a possible recent pertussis infection at baseline were excluded from the decay model because of potential recent infection at the start of the study (diagnostic criterion based on a single serum/plasma sample) [28]. The final datapoints from participants who became infected during the study and participants who received an extra vaccination during the study (based on the questionnaire) were also excluded from the decay model. Additionally, the last datapoint from participants who showed an increase in antibody concentration between the 1-year timepoint and the last timepoint were excluded from the decay model for the concerning antigen. Although they might not have met the criteria for pertussis infection using a paired serum/plasma sample, natural boosting with *B. pertussis* could not be excluded; an increase in diphtheria toxoid (Dtxd) and tetanus toxin (Ttx) could have been caused by a Dtxd and/or tetanus toxoid (Ttxd) containing vaccination, since this was not specifically asked in the questionnaire.

The proportion of individuals with protective antibody concentrations was calculated based on the decay model per study cohort based on a Ttx and Dtxd IgG-antibody concentration cut-off of 0.1 IU/mL against tetanus and diphtheria respectively, and an arbitrary cut-off of 20 IU/mL for pertussis toxin (Ptx) for the proportion with protective antibody concentrations against pertussis disease [23,24,25,26,27].

Serum/plasma infection prevalence during the study was also studied, including a 2-step risk analysis for contracting pertussis. Step 1: we included all participants to calculate a crude odds ratio to determine whether there were risk factors independently associated with an increased chance of contracting pertussis, using logistic regression analysis. All variables were first tested in a univariate model and variables with a *p* value < 0.1 were included in the multivariable model. By stepwise backward selection, variables independently associated with infection were identified. Step 2: to avoid the influence of age or priming vaccination background, significances found in the multivariable model were analysed using an appropriate statistical test comparing two out of three cohorts: either the acellular pertussis (aP) primed children to the whole cell pertussis (wP) primed children or the wP-primed children to the wP-primed adults.

## 3. Results

### 3.1. Geometric Mean Antibody Concentrations (GMCs) per Timepoint

To investigate differences in the long-term immunogenicity of an aP booster between age groups and vaccination backgrounds, we measured antibody GMCs in aP-primed children, wP-primed children, and wP-primed adults at baseline, 28 days, 1 year, and approximately 6 years post the aP booster vaccination. For adults, there were additional timepoints at 14 days and 2 years post the aP booster vaccination. Antibody GMC data are presented in Table 1 and the individual responses in Figure S3. Antibody concentrations in adults were highest 14 days post-vaccination for all antigens. Children showed the highest antibody concentrations 28 days post-vaccination; no blood was drawn at day 14. Approximately 6 years post-vaccination, GMCs still tended to be higher compared with baseline in all three study groups. Infection during the study was defined as a three-fold increase in IgG-Ptx between the one-year timepoint and the last follow-up timepoint after approximately 6 years, combined with IgG-Ptx ≥20 IU/mL at the last timepoint. Based on this definition, 12% (7/59) of the aP-primed paediatric cohort contracted pertussis between 1 year and 6–7 years post the booster vaccination in contrast to 2% (1/49) in the wP-primed paediatric cohort between 1 year and 5–6 years post the booster vaccination. No adults met the criterion for pertussis infection between 1 year and 6 years post the booster vaccination, while 21% (18/85) of adults received an extra pertussis vaccination during the study (in addition to the study intervention). This included vaccination during pregnancy, or incidentally was as a work-related requirement. In both paediatric cohorts, no additional pertussis vaccinations were reported, though in contrast to adults, all had been routinely aP-boosted at 4 years of age.

### 3.2. Antibody Kinetics

**Figure 2:**
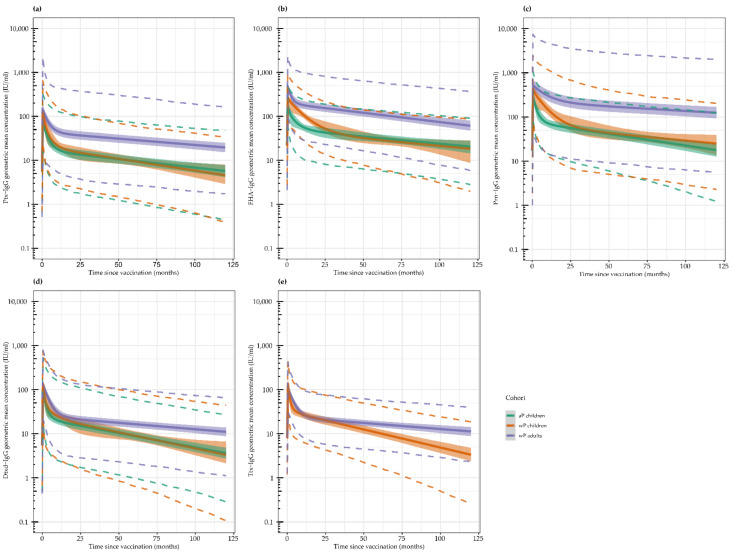
Modelled IgG antibody concentrations from time of vaccination (0 months) up to 10 years (120 months) post-vaccination in acellular pertussis (aP) primed children, whole cell pertussis (wP) primed children, and wP-primed young adults, as the colours in the legend indicate. Modelled geometric mean IgG concentrations are shown (solid lines) with their respective 95% confidence interval (ribbons). Dashed lines show the range where 95% of most probable predictions lie. Datapoints underlying this model can be viewed in Supplementary Figure S2. Acellular primed children are missing in the tetanus toxin model because of interference from another (non-pertussis) vaccine in between the last two timepoints. IU: international units; Ptx: pertussis toxin; FHA: filamentous haemagglutinin; Prn: pertactin; Dtxd; diphtheria toxoid; Txt: tetanus toxin; IgG: immunoglobulin G. Modelled IgG antibody concentrations for (**a**): pertussis toxin, (**b**): filamentous haemagglutinin; (**c**): pertactin; (**d**): diphtheria toxoid; and (**e**): tetanus toxin.

In Figure 2, we present model predicted antibody concentrations up to 10 years (120 months) post-vaccination for the five vaccine antigens. This was based on the majority of the participants’ individual datapoints over time until approximately 6 years post-vaccination, as presented in Figure S3. For all antigens, we observed an increase in antibody concentrations caused by vaccination which peaked within the first month post-vaccination, followed by a biphasic antibody decay with a rapid decline in the first phase, as described in previous studies, with 1-year follow-up and a slower decline in the second phase between 1 year and 6 years [17,18,19].

For Ptx, the initial peak was higher in wP-primed children and adults compared with aP-primed children (Figure 2a). The initial decline in both wP- and aP-primed children started early and resulted in similar antibody concentrations and antibody kinetics in both groups within a year after vaccination. Adults tended to have a slower decline in the second phase leading to a higher upkeep of Ptx antibodies over time, however, the distribution of antibody concentrations was more heterogeneous in adults at all times compared to children.

For FHA, the highest peak in antibody concentrations was seen in wP-primed adults, followed by wP-primed children, and was lowest in aP-primed children (Figure 2b). Antibody kinetics for wP- and aP-primed children were initially different. Children with a wP-priming background tended to have a slower initial decline, but the duration of decline seemed to be over a longer period compared with aP-primed children, resulting in similar concentrations between both groups from approximately 30 months (2.5 years) post the booster vaccination onwards. Adults had similar FHA antibody kinetics compared to aP-primed children, but since they started with higher peak concentrations, their antibody concentrations remain higher for a longer period.

The Prn antibody peak concentrations were similar for the three groups, but the initial and second decay phases were much slower in the adults compared to both paediatric groups (Figure 2c). Similar to the responses against FHA, the initial decline in Prn-specific antibody concentrations in wP-primed children was slower compared to aP-primed children with a longer duration, resulting in similar kinetics between wP- and aP-primed children from approximately 30 months (2.5 years) post-vaccination onwards.

The peak concentrations for Dtxd antibodies were slightly higher in adults, but the main difference between wP-primed adults and both paediatric groups is the extremely slow second decay phase leading to higher upkeep of antibodies in adults over time (Figure 2d).

For Ttx, antibody concentrations of only the wP-primed adults and children were compared since the aP-primed children received a Ttxd conjugated meningococcal vaccine during the study, which boosted their immune response to Ttxd (Figure 2e). Antibody decay differences between the adults and the children were similar to that of Dtxd, again showing an extremely slow second decay phase in the adult cohort compared to the children.

### 3.3. Proportion with Protective Antibody Concentrations

**Figure 3:**
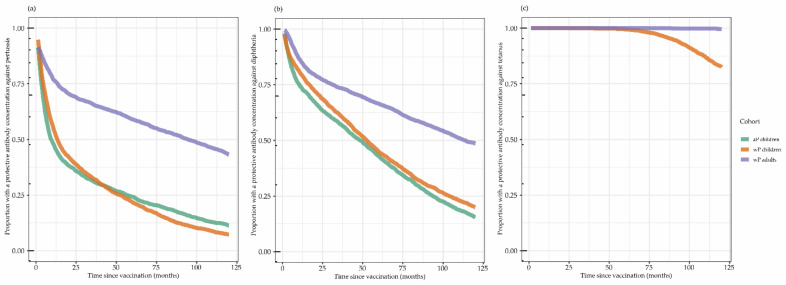
The modelled proportion with protective antibody concentrations is based on the antibody decay model (Figure 2). For pertussis toxin, an arbitrary cut-off of protection of ≥20 IU/mL was used. For both diphtheria toxoid and tetanus toxin, the WHO cut-off for clinical protection of 0.1 IU/mL was used. Acellular primed children are missing in the tetanus toxin model because of interference from another (non-pertussis) vaccine in between the last two timepoints. aP: acellular pertussis; wP: whole cell pertussis. The modelled proportion with protective antibody concentrations for (**a**): pertussis; (**b**): diphtheria; and (**c**): tetanus.

Figure 3 shows the modelled proportion of individuals protected against pertussis, diphtheria, and tetanus per cohort. The presumed protection against clinical pertussis (arbitrary cut-off of IgG-Ptx ≥20 IU/mL) over 10 years (120 months) is illustrated in Figure 3a. The height of the peak concentration as well as the speed of the initial fast decay and the second slow decay phase influenced the proportion of participants with protective antibody concentrations at a certain timepoint. This resulted in 50% of children with antibody concentrations already below the limit of protection against clinical pertussis in both paediatric cohorts around 1 year (12 months) post-vaccination, whereas in the wP-primed adults this 50% point was only reached around 7.5 years (90 months) post-vaccination.

The Dtxd antibodies illustrated in Figure 3b wane more slowly compared to the Ptx antibodies, and 50% of children (both aP- and wP-primed) were clinically protected (IgG-Dtxd ≥ 0.1 IU/mL) for approximately 4 years (48 months), while adults reach this 50% protection point after approximately 9 years (108 months).

The Ttx antibodies illustrated in Figure 3c barely declined in adults and started to decline after 5 years (60 months) in wP-primed children, resulting in almost 100% of adults still protected even after 10 years (120 months) and more than 75% of wP-primed children still protected. From the decay model, it can be calculated that the GMC of the wP-primed children reached the limit of clinical protection (IgG-Ttx ≥ 0.1 IU/mL) after 14 years and for adults only after 38 years.

### 3.4. Risk Factors to Contract Pertussis

From the questionnaires, it appeared that there were no clinically symptomatic cases diagnosed as pertussis reported in any of the three cohorts during approximately 6 years follow-up time. However, there appeared to be multiple participants who showed a three-fold increase in IgG-Ptx antibodies reaching at least 20 IU/mL during the study, pointing to pertussis infection according to the Dutch criterion based on a paired serum/plasma sample. Based on this definition, one participant became infected in the cohort of wP-primed children in contrast to seven in the cohort of aP-primed children, and no adults became infected. In Table 2, we performed a 2-step risk analysis. In step 1, a number of risk factors for contracting pertussis is listed and was used for logistic regression analysis on the entire cohort. Univariately tested aP priming, Ptx antibody concentrations <20 IU/mL at 1 year post-vaccination, and low FHA antibody concentrations 1 month post-vaccination predicted a higher risk of contracting pertussis. Using the multivariable model, only the type of priming vaccination was significantly associated with periods of pertussis infection. Individuals primed with aP vaccines in infancy had 11 times higher odds of contracting pertussis compared to wP-primed individuals. In step 2, we used a Pearson Chi-Square test to compare the proportion of infected children in the aP-primed cohort with the proportion of infected children the wP-primed cohort. Adults were not included in this analysis to avoid the influence of age. The risk of contracting pertussis almost reached significance (*p* = 0.052), though we cannot correct for differences in the follow-up time and the degree of exposure to *B. pertussis*.

## 4. Discussion

We set out to determine the long-term humoral immunity after an aP booster vaccination in both children and adults and compared the initial priming vaccinations in infancy in children. We found that pertussis antibody concentrations after an aP booster vaccination are higher in adults compared with children and antibody concentrations persist at a higher level for over a decade. Serological evidence of recent pertussis infection was more often found after aP-priming vaccinations in infancy compared with wP priming vaccinations.

Differences in antibody kinetics between wP-primed children and wP-primed adults with ultimately higher antibody concentrations in the young adults, are probably caused by the exposure to *B. pertussis* during life. Cumulative local pertussis disease notification data obtained from the RIVM indicates that the degree of exposure to *B. pertussis* during the study was highest in the wP-primed children cohort and lowest in the wP-primed adults. Nevertheless, the young adults had a longer life with more opportunity to become infected considering the high pertussis infection prevalence in the Netherlands [34]. These possible infections are likely to have boosted their immune systems and might explain the slower waning in antibodies in the young adults. The slow waning in Ptx antibody concentrations in adults has been previously described in a Danish study by Dalby et al. where they used a study vaccine that contained 20 µg Ptx, instead of 8 µg as in our study [35]. In contrast, Pool et al. observed fast waning in Ptx antibody concentrations in adolescents as well as in adults that were already almost back to pre-vaccination levels 5 years post-vaccination in a study in the USA [15]. From this we can assume that the antibody decay rate is multifactorially determined. The dose of the booster vaccine, but possibly also the dose and number of previous vaccinations, in combination with the frequency of natural boosting, will probably influence the antibody response and the decay rate.

Antibodies rapidly waned in the wP-primed paediatric cohort with ultimately comparable kinetics to aP-primed children. However, most of the wP-primed children, unlike aP-primed children, appeared to still be protected against infection during the follow-up, based on the serological defined cut-off of infection. In the aP-primed paediatric cohort, 12% met our serological criterion of pertussis infection after six years compared with 2% in the wP-primed paediatric cohort. The observed difference in the proportion of pertussis infected individuals in aP-primed children compared to the wP-primed children is close to significance (*p* = 0.052). This is in line with findings described in non-human primates, where aP priming compared with wP priming led to higher infectious susceptibility [36]. Therefore, protection in the long term is likely to be dependent not only on humoral immunity but also on cellular immunity, which has been suggested before, however, a correlate of protection against pertussis has not yet been established [37]. Previous reports regarding our two paediatric cohorts showed that aP-primed children had a higher proportion of IgG4 antibodies following aP boosting, indicating more Th2 skewing compared with wP-primed children [38,39]. One year post-booster vaccination, the difference in the proportion of IgG4 still existed. The higher proportion of IgG4 might be caused by the relatively lower T helper (Th) 1/Th2 ratio in aP-primed children [39]. A Th2-skewed response has frequently been described in aP-primed individuals in contrast to a more Th1/Th17-skewed immune response in wP-primed individuals [18,40]. In baboon studies, it has been shown that Th2 responses protect against clinical disease, but Th1 and Th17 responses are needed to protect against colonisation and transmission [36]. The Th2 skewing in our aP-primed children in combination with rapidly waning antibodies after the booster might have increased their vulnerability to infection.

A limitation of the study is the fact that other aspects may have affected the number of pertussis infections, such as a longer observation period in the aP-primed children compared to the wP-primed children (13 months later) and wP-primed adults (6 months later) (Figure S1). Humoral and cellular immunity may have waned more in the aP-primed paediatric cohort after a longer time compared to the wP-primed cohorts, possibly resulting in higher susceptibility to infection. We cannot discern the time of infection, either early or later, after the aP booster since we have only a single blood sample at the end of the follow-up. Furthermore, the cyclic pattern of pertussis outbreaks as well as the studies not being executed in the same period nor in the same municipalities, may have caused differences in pertussis exposure among the cohorts. In 2012 and 2014, the Netherlands experienced pertussis outbreaks where most disease cases were observed in adolescents [22]. Cumulative local pertussis disease notification data obtained from the RIVM indicates that exposure to *B. pertussis* during the study period was highest in the wP-primed children cohort, which is expected considering the most recent outbreaks, followed by the aP-primed children, and was lowest in the wP-primed adults. Therefore, the degree of pertussis circulation is unlikely to explain the serological differences associated with the proportion of infected participants in the three study cohorts. Taken together, not only priming vaccination background but potentially also in combination with the longer post-booster follow-up time, may have resulted in a higher proportion of infected individuals in the aP-primed paediatric cohort.

Some of the individuals that are aP-primed might form a reservoir for *B. pertussis* and transmit the bacterium to others. Since the proportion of aP-primed individuals in the population is increasing, additional population-wide boosters are unlikely to substantially increase the overall herd immunity to pertussis. If herd immunity is insufficient to achieve protection to vulnerable individuals such as infants and other risk groups such as individuals with pulmonary co-morbidity and older adults, these risk groups might benefit from an extra aP booster at the individual level [2]. Furthermore, certain groups who are prone to transmit infection, such as health care personnel and professionals working with very young children or vulnerable older adults, might benefit from an extra booster [36]. Since most professionals in the Netherlands are currently still wP-primed in infancy, an aP booster may offer reduced transmission by protecting professionals against infection. However, little is known regarding repeated boosters and their advantages or disadvantages [41].

Although some individuals in our study became infected, we had no reported pertussis cases in any of our cohorts. In the Netherlands, the number of reported cases in adolescents and young adults is quite low [6,34]. This might be an underestimation because pertussis generally presents less severe symptoms in adolescents and adults [42]. From a nationwide serosurveillance study in the Netherlands we know that serum infection prevalence in the studied age groups is approximately 200 times higher compared to the number of reported cases. Considering that only eight individuals in total became infected, it was not surprising that we did not find any symptomatic pertussis cases. Therefore, we cannot conclude from our data if an aP booster protects against the disease, however, from the literature it is known that aP boosters do protect against the disease in the short term in aP- and wP-primed individuals [43]. In the long term, aP-primed individuals are less protected against the disease compared to their wP-primed peers [44].

Antibody kinetics of our young adult cohort might be translatable to maternal vaccination since Huygen et al. found that pregnant and non-pregnant women respond comparably to an aP booster vaccine up to at least one year post-vaccination [45]. This means that we could expect the same major inter-individual variability in antibody responses between pregnant women as we have seen between our young adult participants. The current recommendation is to boost expectant women every pregnancy to induce high antibody concentrations necessary for sufficient transplacental transmission [46]. However, considering the slow decline in antibodies, it is questionable if all pregnant women would benefit from a booster at subsequent pregnancies, as some might still have very high antibody concentrations from a previous (maternal) booster. Determination of IgG-Ptx antibody concentrations early in pregnancy for every subsequent pregnancy after the first could help to substantiate whether expectant mothers would benefit from a subsequent booster. Since blood is already drawn early in pregnancy for several other (immunological) measurements such as lupus, this is not difficult to implement in the Netherlands.

Pertussis vaccines are always part of a multi-component combination vaccine, including at least Dtxd and Ttxd. Therefore, not only immunity against pertussis but also immunity against diphtheria and tetanus will be boosted. In line with our data and other studies, a decennial booster in adults seems adequate for diphtheria [47]. However, Ttx antibody concentrations decrease much more slowly and IgG-Ttx concentration seems sufficient for a much longer period. Furthermore, tetanus hyper-immunisation could lead to hypersensitivity responses [48,49]. Our results seem to substantiate a tetanus booster approximately every 35 years for adult individuals who participated in the NIP, in line with Hammarlund et al. who also found protective immunity against tetanus for more than 30 years [50]. Current recommendation in the Netherlands is to boost after possible exposure to tetanus if the last vaccination is more than 10 years ago [51]. Antibody concentrations of Ttx seem to decrease faster among children compared to young adults, and therefore, it seems reasonable to retain a 10-year interval after the booster at 9 years of age before the first possible exposure.

In conclusion, in this phase IV longitudinal interventional study, where we assessed long-term immunity following a pertussis booster vaccination in children and adults, we found heterogeneous but high antibody concentrations in adults. Children primed with aP vaccines had the highest prevalence of serologic parameters indicative of recent pertussis infection. Therefore, an extra aP booster vaccine does not seem to sufficiently protect against infection for more than 6 years after a booster vaccination in aP-primed school aged children. Since the proportion of aP-primed individuals is increasing in the population, it is not likely that the implementation of additional aP boosters after the previous preschool booster would reduce transmission of *B. pertussis*. Protecting risk groups seems most important, with the maternal vaccination as the most essential measure.

## Figures and Tables

**Table 1:** Geometric mean concentrations calculated per cohort per timepoint.

	*n*	GMC Ptx (95% CI)	GMC FHA (95% CI)	GMC Prn (95% CI)	GMC Dtxd (95% CI)	GMC Ttx (95% CI)
Children aP-primed ^1^						
Baseline	83	7 (5–9)	27 (23–33)	36 (29–45)	0.04 (0.03–0.05)	0.35 (0.29–0.44)
28 days	83	68 (57–81)	158 (140–179)	307 (267–352)	0.61 (0.49–0.75)	7.44 (6.31–8.76)
1 year	80	19 (15–24)	60 (50–71)	99 (84–117)	0.15 (0.12–0.19)	1.94 (1.65–2.30)
6 years 9 months	59	14 (10–19)	46 (38–57)	46 (36–60)	0.05 (0.04–0.07)	3.69 (2.95–4.61) *
6 years 9 months: infected	7 (12%)	57 (37–87)	128 (80–202)	84 (22–319)	N/A	N/A
6 years 9 months: non-infected	52 (88%)	12 (8–16)	41 (33–50)	43 (33–55)	N/A	N/A
Children wP-primed ^2^						
Baseline	83	7 (5–10)	34 (26–44)	13 (10–18)	0.05 (0.04–0.06)	0.46 (0.38–0.56)
28 days	81	112 (91–139)	282 (244–325)	343 (275–429)	0.88 (0.69–1.12)	9.10 (7.90–10.5)
1 year	79	24 (18–30)	105 (92–121)	93 (70–124)	0.19 (0.14–0.25)	2.09 (1.78–2.45)
5 years 8 months	49	13 (9–18)	34 (27–43)	34 (24–47)	0.07 (0.05–0.10)	0.66 (0.52–0.82)
5 years 8 months infected	1 (2%)	41 (–)	183 (–)	43 (–)	N/A	N/A
5 years 8 months non-infected	48 (98%)	13 (9–18)	33 (27–41)	33 (23–47)	N/A	N/A
Adults wP-primed ^3^						
Baseline	104	5 (4–7)	11 (8–13)	11 (8–14)	0.09 (0.07–0.11)	1.28 (1.07–1.53)
14 days	103	130 (94–179)	408 (343–485)	369 (277–492)	1.56 (1.30–1.89)	11.7 (10.1–13.5)
28 days	102	122 (93–161)	341 (289–402)	364 (275–483)	1.27 (1.07–1.52)	9.44 (8.36–10.7)
1 year	100	43 (33–55)	133 (110–160)	186 (136–253)	0.35 (0.29–0.43)	2.97 (2.63–3.34)
2 years	99	34 (26–44)	109 (90–132)	146 (107–199)	0.24 (0.20–0.29)	2.15 (1.90–2.43)
6 years 3 months	85	32 (25–43)	72 (59–89)	132 (97–180)	0.23 (0.19–0.29)	2.47 (2.13–2.86)
6 years 3 months: extra vaccination	18 (21%)	84 (48–146)	131 (92–187)	214 (114–401)	0.38 (0.22–0.65)	3.69 (2.86–4.77)
6 years 3 months: no extra vaccination	67 (79%)	25 (19–34)	62 (49–78)	116 (81–166)	0.20 (0.17–0.25)	2.21 (1.88–2.61)

If relevant, the group was additionally split up to represent infected individuals and/or participants who received an extra vaccination during the study in addition to the study intervention. ^1^ aP priming vaccinations at 2, 3, 4, and 11 months of age and aP-boosted at 4 years of age. ^2^ wP priming vaccinations at 2, 3, 4, and 11 months of age and aP-boosted at 4 years of age. ^3^ wP priming vaccinations at 3, 4, 5 and 11 months of age, no preschool booster. * Antibody concentrations increased because of meningococcal A, C, W, Y catch-up vaccination as the concerning vaccine contained tetanus toxoid conjugate. n: number; GMC: geometric mean concentration, Ptx: pertussis toxin; CI: confidence interval; FHA: filamentous haemagglutinin; Prn: pertactin; Dtxd: diphtheria toxoid; Ttx: tetanus toxin; aP: acellular pertussis; wP: whole cell pertussis; N/A: not applicable.

**Table 2:** Risk factors for contracting pertussis.

	*n* (%)	N Recent Pertussis Infection (%)	Univariate Crude OR (95% CI)	*p*-Value	Multivariate Adjusted OR (95% CI)	*p*-Value
Step 1: multivariable model with all participants combined						
Sex				0.322		
Male	79 (45%)	5 (6.3%)	Ref.			
Female	96 (55%)	3 (3.1%)	0.477 (0.110–2.063)			
Age at inclusion				0.997		
9 years	108 (62%)	8 (7.4%)	Ref.			
25–29 years	67 (38%)	0 (0.0%)	<0.001 (N/A)			
Priming vaccinations				0.011		0.028
wP	116 (66%)	1 (0.9%)	Ref.		Ref.	
aP	59 (34%)	7 (12%)	15.481 (1.857–129.067)		11.061 (1.293–94.604)	
Ptx < 20 IU/mL at 1 year				0.025		0.063
no	102 (59%)	1 (1.0%)	Ref.		Ref.	
yes	70 (41%)	7 (10%)	11.222 (1.349–93.380)		7.701 (0.895–66.294)	
Antibody concentrations one moths post-vaccination						
Ptx Ab at 1 month *	173	8 (4.6%)	0.440 (0.123–1.577)	0.208		
FHA Ab at 1 month *	173	8 (4.6%)	0.069 (0.005–0.877)	0.039		
Prn Ab at 1 month *	173	8 (4.6%)	0.618 (0.146–2.627)	0.515		
Ptx Ab at 1 year *	172	8 (4.7%)	0.316 (0.087–1.146)	0.080		
FHA Ab at 1year *	172	8 (4.7%)	0.088 (0.011–0.736)	0.025		
Prn Ab at 1 year *	172	8 (4.7%)	0.582 (0.168–2.019)	0.148		
Step 2: Pearson’s chi-squared test on priming vaccinations between aP- and wP-primed children (adults excluded)						
wP-primed children	49 (45%)	1 (2.0%)	N/A	0.052		
aP-primed children	59 (55%)	7 (12%)				

* Added as a continuous variable. n: number; OR: odds ratio; CI: confidence interval; aP: acellular pertussis; wP: whole cell pertussis; Ptx: pertussis toxin; FHA: filamentous haemagglutinin; Prn: pertactin; Ab: antibody concentrations; N/A: not applicable.

## Data Availability

The data presented in this study are available upon request from the corresponding author.

## Supplementary Materials

The following supporting information can be downloaded at: https://www.mdpi.com/article/10.3390/vaccines10050693/s1, Figure S1: Timelines for the different study cohorts; Figure S2: Cumulative local pertussis disease notifications; Figure S3: Individual antibody concentrations; Table S1: *B. Pertussis* vaccine schedules and vaccines in the Netherlands.
