# Supplementary material for: Long-Term Immunogenicity upon Pertussis Booster Vaccination in Young Adults and Children in Relation to Priming Vaccinations in Infancy
Source: Vaccines (Basel). 2022 Apr 28;10(5):693. doi: 10.3390/vaccines10050693 (PMC9146390; doi:10.3390/vaccines10050693)
Supplement: Supplementary file 1 [file vaccines-10-00693-s001.zip › vaccines-1684291-supplementary.pdf]

vaccines

Supplementary Materials

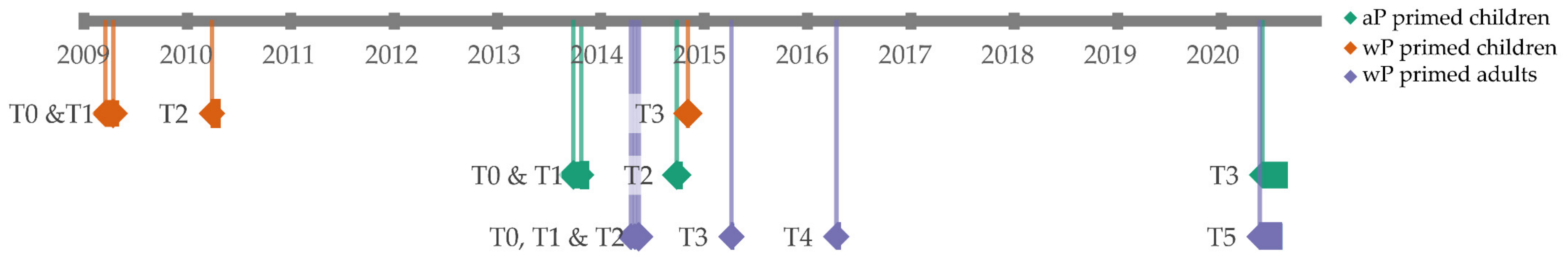

Figure S1. Timelines for the different study cohorts. T0-T5 represent the study visits; aP primed children were recruited in the municipalities of Almere, Breda, Eindhoven, Ede, and Utrecht; wP primed children were recruited in the city of Hoofddorp; wP primed adults were recruited in the municipality of Amersfoort. aP: acellular pertussis; wP: whole cell pertussis ; T: time point.

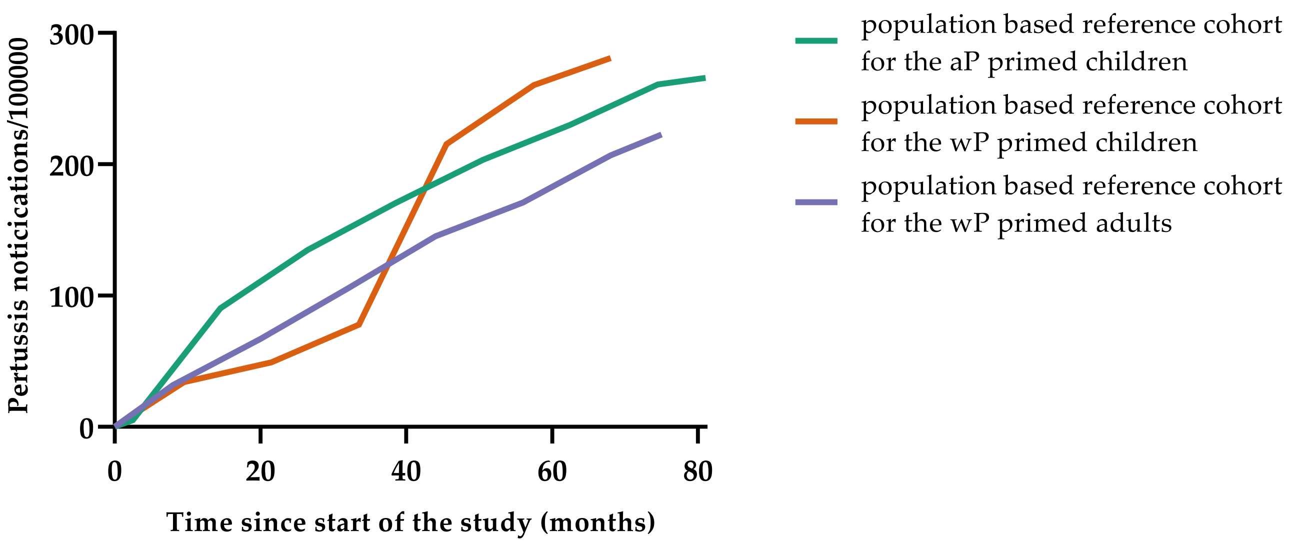

Figure S2. Cumulative local pertussis disease notifications per 100,000 inhabitants. Pertussis disease notification data were obtained from the Dutch National Institute for Public Health and the Environment (RIVM) based on the postal code matched regions at times of inclusion for the three study cohorts, covering the period from inclusion to final blood draw per cohort. Incidence is plotted against the amount of inhabitants in the concerning region. aP: acellular pertussis; wP: whole cell pertussis

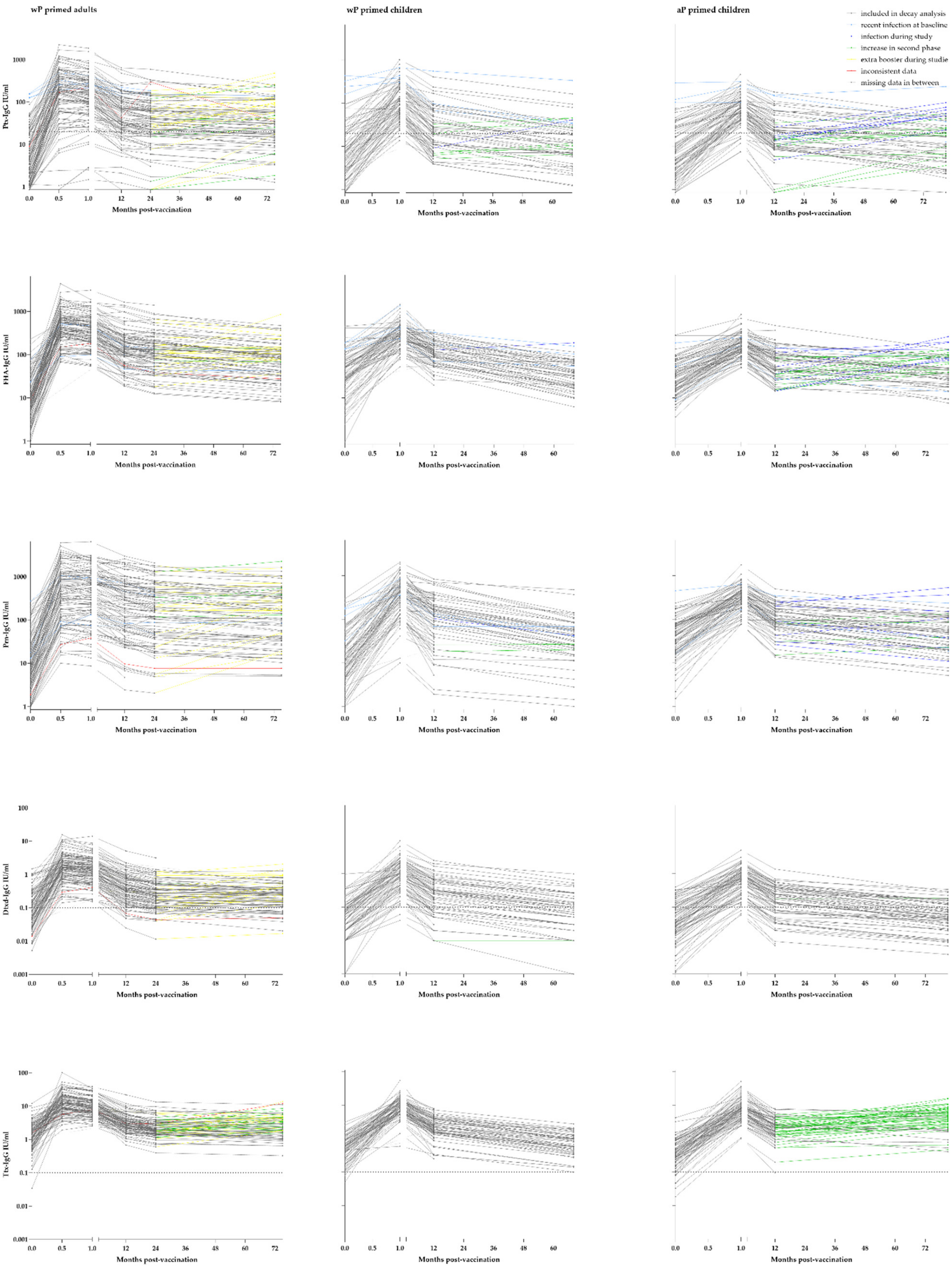

Figure S3. Individual responses. aP: acellular pertussis; wP: whole cell pertussis IU: international units; Ptx: pertussis toxin; FHA: filamentous haemagglutinin; Prn: pertactin; Dxtd; diphtheria toxoid; Txt: tetanus toxin.

Table S1. *B. Pertussis* vaccine schedules and vaccines in the Netherlands.

Year	Scheme	Vaccine composition	Change	pertussis Vaccine name (Company)
before				No vaccine available
1954		DTwP	Combination vaccine became available	DTwP (RIVM)
1957	3,4,5,11 m	DTwP	Start of NIP	DTwP (RIVM)
1962	3,4,5,11 m	DTwP-IPV	Addition of IPV	DTwP-IPV (RIVM)
1997	3,4,5,11 m	DTwP-IPV + Hib	Addition of Hib tot schedule	DTwP-IPV (RIVM)
1999	2,3,4,11 m	DTwP-IPV + Hib	Accelerated vaccination	DTwP-IPV (RIVM)
2001	2,3,4,11 m	DTwP-IPV + Hib		DTwP-IPV (RIVM)
	4y	DT-IPV + aP3	Introduction aP booster, 3 Bp components	monovalent aP (GSK)
2003	2,3,4,11 m	DTwP-IPV-Hib	Combination vaccine	DTwP-IPV/Hib (NVI)
	4y	DT-IPV + aP3		monovalent aP (GSK)
2005	2,3,4,11 m	DTaP3-IPV-Hib	Introduction aP priming, 3 Bp components	Infanrix IPV + Hib (GSK)
	4y	DT-IPV + aP3		monovalent aP (GSK)
2006 (Jan)	2,3,4,11 m	DTaP5-IPV-Hib	5 Bp components	Pediacel (SP MSD)
	4y	DT-IPV + aP3		monovalent aP (GSK)
2006 (Jun)	2,3,4,11 m	DTaP5-IPV-Hib + Pneu	Addition of Pneu to schedule;	Pediacel (SP MSD)
	4y	DT-IPV + aP3		monovalent aP (GSK)
2006 (Jul)	2,3,4,11 m	DTaP5-IPV-Hib + Pneu		Pediacel (SP MSD)
	4y	DTaP5-IPV	Combination vaccine, 5 Bp components	Triaxis Polio (SP MSD)
2008 (Feb)	2,3,4,11 m	DTaP5P-IPV-Hib + Pneu		Pediacel (SP MSD)
	4y	DTaP3-IPV	3 Bp components	Infanrix-IPV (GSK)
2008 (Jul)	2,3,4,11 m	DTaP3-IPV-Hib + Pneu	3 Bp components	Infanrix-IPV + Hib (GSK)
	4y	DTaP3-IPV		Infanrix-IPV (GSK)
2009	2,3,4,11 m	DTaP3/5-IPV-Hib + Pneu	3 or 5 Bp components	Pediacel (SP MSD)/Infanrix-IPV + Hib (GSK)
	4y	DTaP3-IPV		Infanrix-IPV (GSK)
2010	2,3,4,11 m	DTaP5-IPV-Hib + Pneu	5 Bp components	Pediacel (SP MSD)
	4y	DTaP3-IPV		Infanrix-IPV (GSK)
2011 (Oct)	2,3,4,11 m	DTaP3-IPV-Hib-HepB + Pneu	Addition of HepB to schedule, 3 Bp components	Infanrix hexa (GSK)
	4y	DTaP3-IPV		Infanrix-IPV (GSK)
2017 (Jan)	2,3,4,11 m	DTaP3-IPV-Hib-HepB + Pneu		Infanrix hexa (GSK)
	4y	Tdap3-IPV	Reduced dose Bp components	Boostrix-IPV (GSK)
2018 (Dec)	2,3,4,11 m	DTaP5-IPV-Hib-HepB + Pneu	5 Bp components	Vaxelis (SP MSD)
	4y	Tdap3-IPV		Boostrix-IPV (GSK)
2019 (Dec)	Maternal (22 weeks gestational age)	Tdap3	Booster for pregnant women, 3 reduced dose Bp components	Boostrix (GSK)
2020	3, 5, 11m	DTaP3-IPV-Hib-HepB + Pneu	Revised priming schedule	Vaxelis (SP MSD)
	4y	Tdap3-IPV		Boostrix-IPV (GSK)

Supplemantary Table 1. m=months; y= years; D=diphtheria; T=tetanus; wP: whole cell pertussis; aP: acellular pertussis; IPV: inactivated poliovirus; Pneu: pneumococcal; Hib: Haemophilus influenzae type B; HepB: Hepatitis B; Bp: *Bordetella pertussis*; NVI: Netherlands Vaccine Institute; GSK: GlaxoSmithKline; SP: Sanofi Pasteur; MSD: Merck Sharp & Dohme. This table is a modified from Versteegen P, Berbers GAM, Smits G, et al. More than 10 years after introduction of an acellular pertussis vaccine in infancy: a cross-sectional serosurvey of pertussis in the Netherlands. *The Lancet Regional Health – Europe* 2021; **6**(10): 1016. doi: 10./j.lanepe.2021.100196.
